# Highly ordered arrays of hat-shaped hierarchical nanostructures with different curvatures for sensitive SERS and plasmon-driven catalysis

**DOI:** 10.1515/nanoph-2021-0476

**Published:** 2021-11-15

**Authors:** Chao Zhang, Zhaoxiang Li, Si Qiu, Weixi Lu, Mingrui Shao, Chang Ji, Guangcan Wang, Xiaofei Zhao, Jing Yu, Zhen Li

**Affiliations:** School of Physics and Electronics, Shandong Normal University, Jinan 250014, China

**Keywords:** hat-shaped nanostructures, SERS, surfacecatalyticreaction

## Abstract

Regulation of hot spots exhibits excellent potential in many applications including nanolasers, energy harvesting, sensing, and subwavelength imaging. Here, hat-shaped hierarchical nanostructures with different space curvatures have been proposed to enhance hot spots for facilitating surface-enhanced Raman scattering (SERS) and plasmon-driven catalysis applications. These novel nanostructures comprise two layers of metal nanoparticles separated by hat-shaped MoS_2_ films. The fabrication of this hybrid structure is based on the thermal annealing and thermal evaporation of self-assembled polystyrene spheres, which are convenient to control the metal particle size and the curvature of hat-shaped nanostructures. Based on the narrow gaps produced by the MoS_2_ films and the curvature of space, the constructed platform exhibits superior SERS capability and achieves ultrasensitive detection for toxic molecules. Furthermore, the surface catalytic conversion of p-nitrothiophenol (PNTP) to p, p′-dimercaptobenzene (DMAB) was *in situ* monitored by the SERS substrate. The mechanism governing this regulation of hot spots is also investigated via theoretical simulations.

## 1 Introduction

Raman spectra are generated by inelastic light scattering caused by molecular vibrations and can offer fingerprint information for molecular analysis [1]. However, the intensity of the scattered light is only about 10^−10^ of the incident light intensity, which significantly limits the scope of applications. Surface-enhanced Raman scattering (SERS) not only inherits the advantages of Raman spectroscopy, but also realizes great signal enhancement; therefore, it is a powerful trace detection tool that can achieve single-molecule level detection [2]. To date, SERS has been applied in various applications including food testing, pharmaceutical analysis, and pollution monitoring [3], [4], [5]. Currently, SERS is known to originate from two mechanisms: electromagnetic mechanism (EM) and chemical mechanism (CM); EM is generally believed to play a major role [6, 7]. The plasmonic metals usually Ag, Au, and Cu, possess the abundant loosely bound electrons that would oscillate in resonance under the exciting radiation (SPR). Owing to the EM that originates from the SPR of metal nanostructures, hot spots are formed at the nanoscale gaps among metal nanostructures and produce huge electromagnetic field enhancement. Generally, the intensity of hot spots increases with a decrease in the gap distance, and the electric field enhancement factor (EF) generally exceeds 10^7^ [8, 9]. The CM is another important factor based on the charge transfer between the molecule and substrate; it can regulate the electron density distributions of molecules, causing great degree of polarization, and enhancing the Raman signals [10].

Various SERS substrates have been proposed to achieve a large EF, including 0D single isolated nanospheres, 1D nanowires, nanorods, and 2D/3D nanoarrays [11], [12], [13], [14], [15]. The poor structural maneuverability of low-dimensional metal nanostructures limits the generation of high-density hot spots, which restricts further improvement of the sensitivity of SERS. Compared with the low-dimensional mode, the 3D metal nanostructure possesses better optical tunability and has attracted wide attention in the field of SERS sensing. Generally, the local electric field enhancement of hot spots is extremely sensitive to the interval size (<≈5 nm) among the metal nanostructures. Numerous works have fabricated 3D SERS substrates with nanoscale or sub-nanoscale spacers, which significantly enhanced Raman signals [16], [17], [18], [19], [20]. Wei et al. proposed a copper film system with graphene as the nanospacer by directly placing the copper nanoparticles on the copper surface tightly coated by a graphene film [16]. The EF of this substrate reached 1.89 × 10^7^ for copper phthalocyanine molecules. Wallace Choy from the University of Hong Kong and Sun Jie from Chalmers University of Technology used the finite-difference time-domain method to investigate the electromagnetic field distribution of two composite structures: silver nanoparticles (Ag NPs)/graphene/Ag films and gold nanoparticles (Au NPs)/graphene/Au NP hybrid structure; the simulation results indicate that the strongest hot spots exists in the graphene spacer region [17, 18]. Kim et al. reported an Au nanowire/graphene/metal film composite structure, which achieved an EF of 1.18 × 10^6^ with copper phthalocyanine [19]. The coupling structure of Ag NP and Ag nanowires was constructed by Dong-Ho Kim’s team using a nanoscale Al_2_O_3_ film as the interlayer. By comparison, the Raman signals were enhanced by 192 times than that those obtained with simple Ag nanowires [20]. As a typical near-field analysis method, the SERS substrate with 3D nanostructures exhibited a much larger EF for the design of nanoscale hot spots [21]. Recently, to realize a large spatial distribution of the enhanced electric field, far-field scattering effects caused by SPR were introduced to contribute to the total signal enhancement combined with the near-field effect [22], [23], [24], [25]. Yu et al. proposed a composite nanostructure with Ag NPs decorated on the vertically interlaced ZnO nanosheets; this structure provides wide spatial distributions of hot spots and highly sensitive Raman signals [23]. Zhang et al. constructed a “particle in cavity” structure, with Ag NPs being deposited in a nanobowl array using SiO_2_ as the spacer; the EF of this substrate for R6G molecule was 10^9^ [24]. Fratalocchi et al. introduced a similar structure in which Ag NPs were deposited in one single Au nanobowl; they demonstrated that the warped spaces could manipulate hot spots, thereby resulting in broadband enhancements in volume and magnitude [25].

Inspired by the above-mentioned works, highly ordered arrays were proposed that comprised two layered Ag and Au nanoparticles separated by hat-shaped MoS_2_ films (AgNPM-AuH). These structures provide several advantages compared to those prepared in previous works. Firstly, stable oxides like SiO_2_ and Al_2_O_3_ films have served as dielectric spacers between metal nanostructures in previous works [26]. In this study, the stable, uniform, and high-transmittance MoS_2_ films have been directly grown on the bottom layer of Au NPs and used as the nanometer spacer; here, the advantages of CM such as molecular adsorption, fluorescence quenching, and signal uniformity can be applied to the Raman signals [27]. Secondly, the nanoparticle-gap-film model was improved to the nanoparticle-gap-nanoparticle model, which can elevate the density of hot spots [28], [29], [30], [31]. Thirdly, compared to that of the previously reported warped substrates, the curvature of the hat-shaped structure could be well regulated, which could make better use of the far-field effect to strongly localize the electromagnetic waves [32], [33], [34]. The SERS capability of the constructed platforms with different curvatures was investigated and the maximum EF was 1.09 × 10^9^. Furthermore, for the hot electrons generated from plasmonic decay, the plasmon-driven catalytic conversion of PNTP to DMAB was *in situ* monitored to assess the capacity of hot spots. The mechanism governing the hot spot regulation is also investigated via theoretical simulations. Utilizing the hat-shaped spatial geometries and hierarchical nanostructures as degree of freedom in hot spot regulation should pave a new path toward design principles for applications in photocatalysis, nonlinear optics, sensors, and plasmonic lasers.

## 2 Experimental section

### 2.1 Chemicals and materials

Ethanol, acetone, sodium dodecyl sulfate (SDS), rhodamine 6G (R6G), malachite green (MG), crystal violet (CV), silicon (Si) wafers, and polystyrene (PS) spheres were obtained from Ganzhou Mxene Technology Co., Ltd.

### 2.2 Fabrication of regular monolayer PS sphere array

A common technique for interface self-assembly was used to fabricate monolayer hexagonally packed PS spheres on Si substrates [35]. Firstly, appropriate amount of ethanol was added to PS spheres (5 wt% concentration) with diameters of 300, 500, 700, and 900 nm, which were then dropped in the SDS solution to form closely packed PS sphere arrays. The Si wafer was treated with oxygen plasma for 30 min to make the surface hydrophilic. Then, the Si wafer was placed horizontally in water and the PS spheres were transferred to the surface of the Si wafer. Subsequently, the Si wafer coated with the PS sphere arrays was dried at room temperature and heated at 110 °C for 10 min to further fixation.

### 2.3 Fabrication of the AgNPM-AuH SERS substrates

PS sphere arrays were deposited using a percolated Au film of 5 nm thickness via thermal evaporation [36] at the pressure of 5.5 × 10^−5^ Pa and the deposition rate of approximately 0.5 Å/s. Ammonium tetrathiomolybdate ((NH_4_)_2_MoS_4_, purity 99.99%; 0.01 g) was added to 1 mL of ethylene glycol solution (1 wt%) and subjected to an ultrasound treatment for 60 min to ensure complete dissolution. (NH_4_)_2_MoS_4_ was solution spin-coated on the Au NP/PS spheres at 3000 rpm for 1 min to form a uniform film. These spheres were placed in the quartz tube at a pressure of 10^−3^ Pa; then, 40 sccm H_2_ was introduced into the quartz tube as the protective gas and the temperature was slowly increased to 500 °C. After increasing the temperature for 90 min, the system was cooled down to room temperature under H_2_ protection, and the MoS_2_ films/Au hat-shaped hybrid structures (M-AuH) were obtained [37]. Subsequently, the percolated Ag film was deposited on the surface of M-AuH via thermal evaporation at a 1 Å/s deposition rate. On this basis, continuous AgNPM-AuH arrays with different diameters were obtained.

### 2.4 Characterization

The substrate morphology and composition were characterized by scanning electron microscopy (SEM, Zeiss Gemini Ultra-55) and energy dispersive X-ray spectroscopy (EDS). The absorption spectra were measured using a UV–vis photometer (UV3600). The SERS performance was assessed using the Raman spectrometer (Horiba HR Evolution) equipped with a 532 nm laser (0.48 mW) at room temperature; a 600 g/mm grating was used, the diameter of laser spot was 1 μm, and the signal accumulation time was 4 s. Subsequently, 2 μL of analyte solution was dropped on the substrate, which was followed by natural drying. The AgNPM-AuH substrate was immersed in the 10^−3^ M PNTP ethanol solution for 90 min and dried in a N_2_ atmosphere after alcohol wash for the plasmon-driven surface-catalyzed reactions.

## 3 Results and discussion

### 3.1 Characterization of the AgNPM-AuH

**Figure 1:**
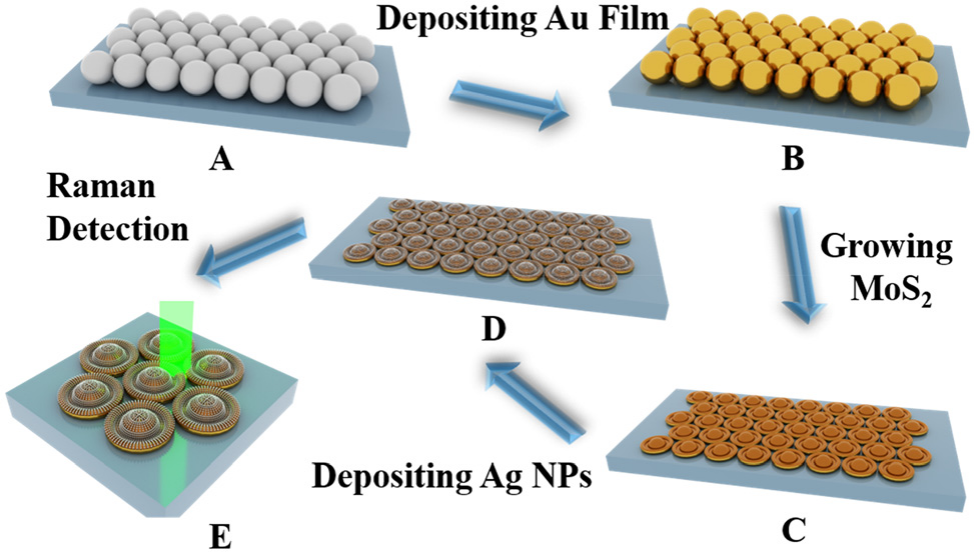
Schematic illustration of the fabrication of hexagonally 3D hat-like AgNPM-AuH arrays: (A) Monolayer of hexagonally close packed PS spheres on Si substrates. (B) Monolayer PS spheres deposited a thin layer of percolated Au film. (C) M-AuH arrays prepared after annealing at 500 °C for 90 min. (D) Hexagonally 3D hat-shaped AgNPM-AuH arrays obtained after the percolated Ag film was deposited. (E) Hexagonally 3D hat-shaped AgNPM-AuH arrays used as SERS substrates.

**Figure 2:**
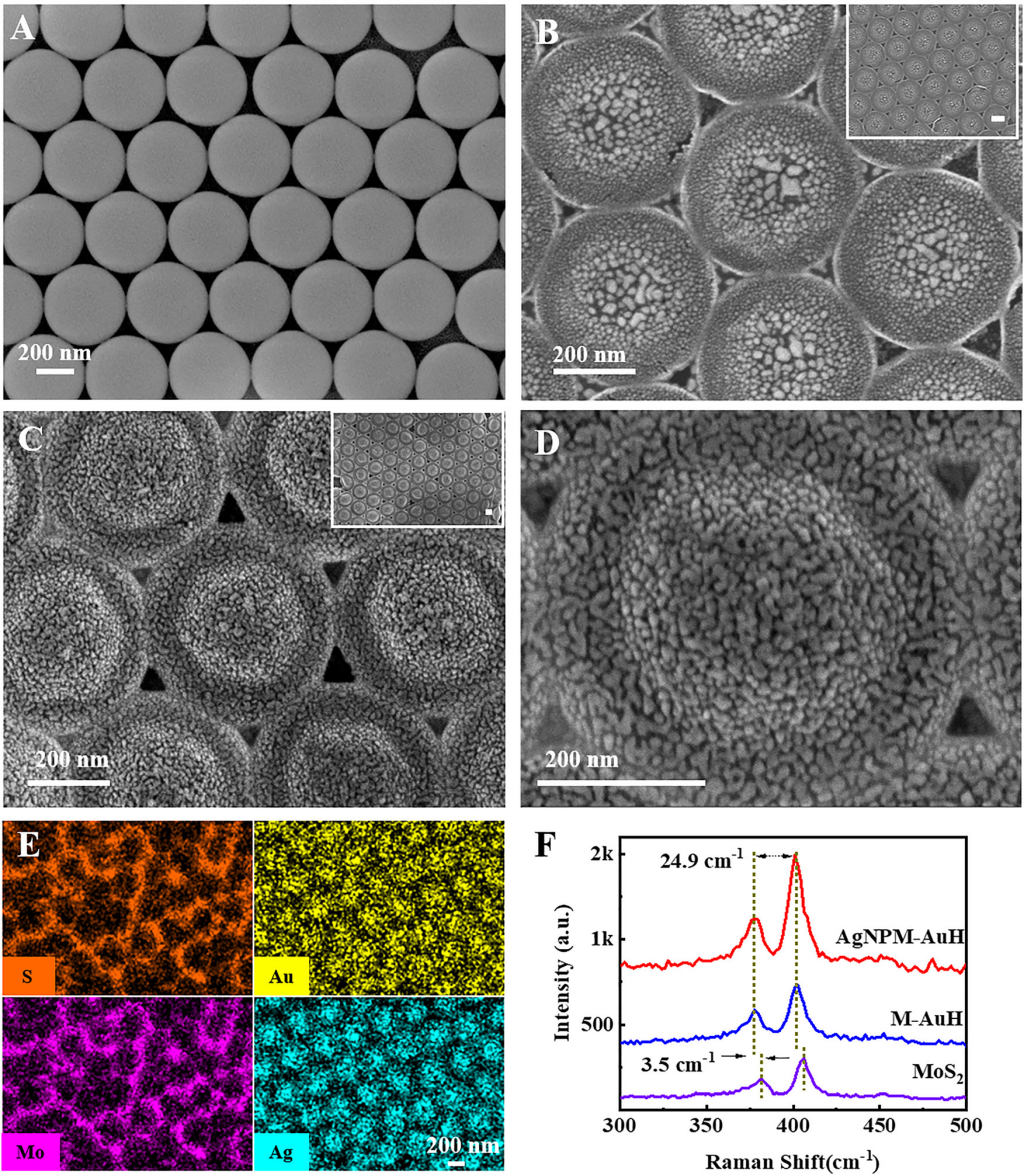
(A) SEM image of hexagonally closely packed PS spheres on Si substrates. (B) SEM image of M-AuH arrays. (C) SEM image of hexagonally 3D hat-shaped AgNPM-AuH arrays. (D) SEM image of a single AgNPM-AuH. (E) Elemental spectrum on the AgNPM-AuH. (F) The Raman spectra of MoS_2_/SiO_2_, M-AuH, and AgNPM-AuH arrays.

The fabrication process of the AgNPM-AuH substrate is illustrated in Figure 1. With the assistance of the template of closely packed PS spheres, the highly ordered AgNPM-AuH arrays were constructed successfully. The detailed process was well presented in the experimental section. The highly ordered AgNPM-AuH arrays were fabricated using the following steps: self-assembly of monolayer PS spheres, MoS_2_ growth, and percolated Ag film deposition. The morphology of the PS spheres plays a prerequisite role for the subsequent transformation. From the SEM image in Figure 2A, we can see that the hexagonally packed PS spheres coated by the percolated Au film with high uniformity were obtained, which is favorable for the reproducibility of Raman signals via SERS application. Conveniently, the PS spheres were evaporated completely in the MoS_2_ growth process due to their thermal instability. Meanwhile, the percolated Au film was also transformed into Au NPs during the process of MoS_2_ growth. The MoS_2_ films are tightly wrapped on the Au NPs, thereby forming the M-AuH core–shell structure shown in Figure 2B . Moreover, the size of the Au NPs was regulated by the thickness of the deposited Au (Figure S1 B–D). Finally, the percolated Ag film was deposited on the M-AuH, and the morphology of the Ag NPs can be adjusted by altering the deposition rate and time of the second metal (Figure S2 A–C). Figure 2C and D exhibit the SEM images of the hexagonal close-packed AgNPM-AuH arrays obtained from the 500 nm PS spheres. The AgNPM-AuH samples obtained from the 300 nm, 700 nm, and 900 nm PS spheres have also been depicted in Figure S3 A–C We note that the AgNPM-AuH morphologies of different sizes are almost the same. Further, there is an annular groove area around the brim, where a large space curvature is formed with two-layered metal nanoparticles; therefore, the regulation of hot spots can be realized based on the space curvature. Here, the MoS_2_ films have three functions: (1) MoS_2_ acts as a framework that retains the hat-shaped structure, which has been proven by the control group shown in Figure S1 A; (2) it can be used as a dielectric nanometer spacer separating the Au and Ag NPs that form high intensity hot spots [38]; (3) The advantages of CM can be utilized for SERS such as molecular adsorption, fluorescence quenching, and signal uniformity [39, 40].

EDS was used to characterize the element composition of AgNPM-AuH. The upper-layered Ag NPs and lower layered Au NPs with highly ordered arrays can be clearly observed in Figure 2E. The uniform distributions of Mo and S prove the presence of MoS_2_ films. Overall, we could make the conclusion that the AgNPM-AuH nanostructures have been successfully fabricated in the experiment. The quality and thickness of the MoS_2_ films has been characterized using the Raman shift (Figure 2F). The frequency difference between the *E* ^1^_2*g*_ and *A*_1*g*_ peaks can be used to estimate the number of MoS_2_ layers. Large and uniform MoS_2_ films were obtained on the percolated Au film by controlling the concentration of (NH_4_)_2_MoS_4_ solution and spin coating rotation speed. The Raman spectrum of MoS_2_ and SiO_2_ growth in the same conditions shows the *E* ^1^_2*g*_ and *A*_1*g*_ peaks at 381.3 and 406.2 cm^−1^ and the M-AuH vibration modes at 377.8 and 400.9 cm^−1^, respectively. The frequency differences close to ≈25 indicate the five-layered MoS_2_ films [41]. The *E* ^1^_2*g*_ band is related to the vibrations in the planes of S and Mo atoms, and the *A*_1*g*_ band represents the out-of-plane vibration of S atoms [42]. The *E* ^1^_2*g*_ band has a strong correlation with the disorder caused by the structural deformation of MoS_2_ and embedded metal NPs, while *A*_1*g*_ is sensitive to the interactions between MoS_2_ and metal NPs [43]. Thus, the curvature of the MoS_2_ films and the lattice strain due to the doping of Au NPs cause the redshift of the two vibrational modes in the M-AuH substrate (Figure 2F) [44, 45]. Furthermore, the signal intensity of M-AuH substrate was enhanced due to the SERS effect induced by the localized surface plasmon resonance (LSPR) of Au NPs. For the AgNPM-AuH substrates, the frequency difference remains unchanged. Although extremely strong hot spots were formed between the Ag and Au NPs, the Raman signal of MoS_2_ was further enhanced.

### 3.2 SERS performance of AgNPM-AuH with deposited metal films of different thicknesses

**Figure 3:**
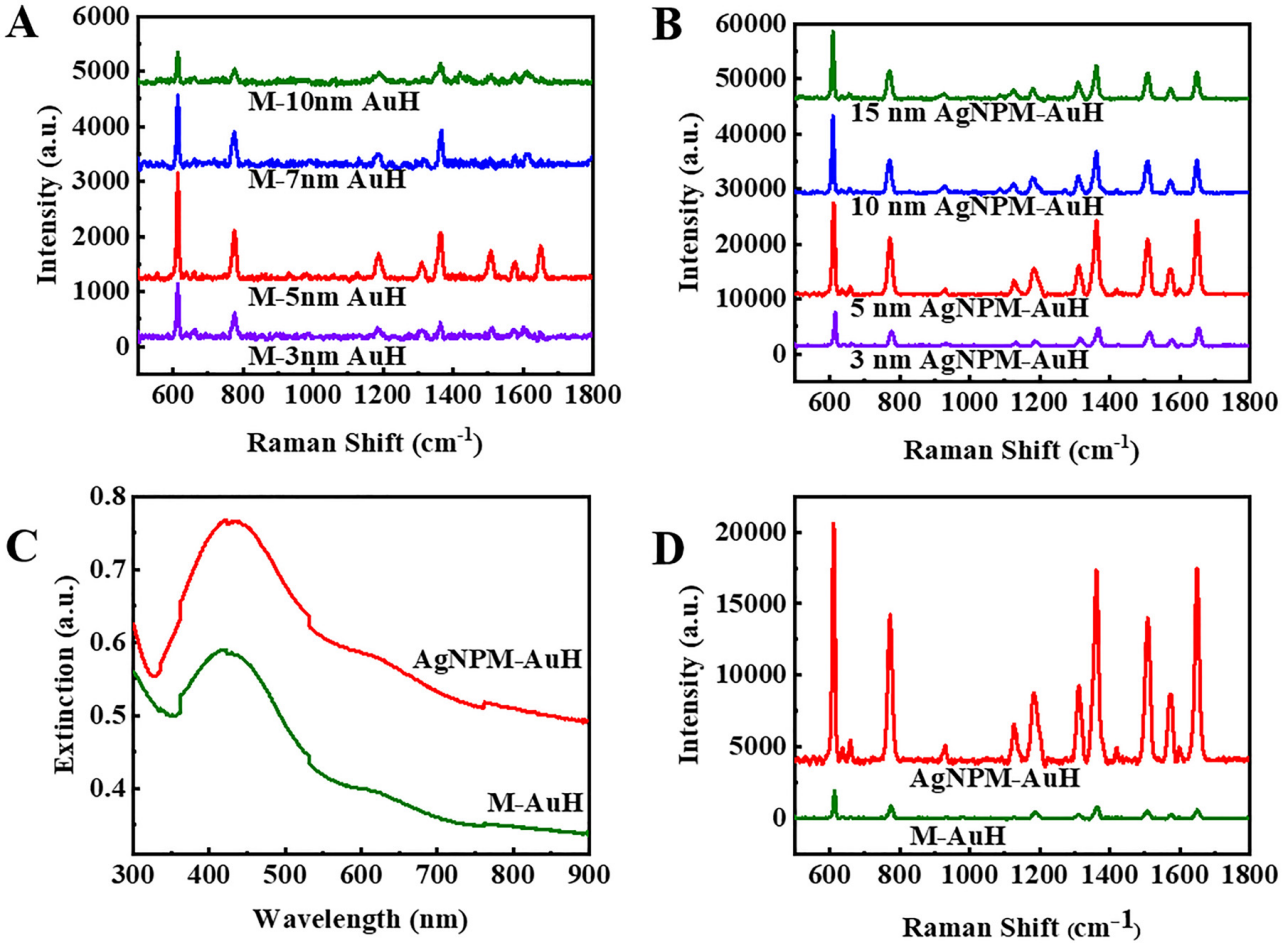
(A) SERS signals of percolated Au film of M-AuH with various thicknesses. (B) Raman spectra of Ag NPs on M-AuH with various thicknesses. (C) Corresponding UV–vis spectroscopy of AgNPM-AuH and M-AuH. (D) Raman spectra of AgNPM-AuH and M-AuH.

Figure S1 B–D exhibit three different morphologies of the M-AuH structures. The size of Au NPs increases as the thickness of the percolated Au film rises, and Au NPs are almost not formed when the percolated Au film with a 10 nm thickness is deposited. The generation of hot spots is related to the gap between Au NPs. We measured the SERS performance of M-AuH substrates by using 10^−6^ M R6G as probe molecules, and the collected SERS signals are shown in Figure 3A. Notably, the SERS signal from M-5-AuH (observed during the fabrication of M-AuH using a 5 nm thick percolated Au film) is much stronger than that observed with other samples, which indicates that a certain thickness of the percolated Au film would generate an appropriate size of Au NPs, a suitable gap in the MoS_2_ growth process, and strong plasmonic couplings [46].

In general, the synergistic effect of all hot spots is responsible for the SERS performance. To obtain a better SERS effect, Ag NPs were deposited. The density of hot spots significantly increases when compared to that of M-AuH. The hot spots distribute not only among the Ag NPs, but also between the Ag and Au NPs. Furthermore, the nanogap created by the MoS_2_ layers lead to larger plasmonic couplings [37]. To test this, the UV–vis spectra of the M-AuH and AgNPM-AuH have been collected in Figure 3C. In the visible wavelength range of 300–900 nm, the AgNPM-AuH substrates show a much stronger light absorption compared to that exhibited by M-AuH, thereby implying a significant improvement in LSPR. The R6G Raman signals collected from M-AuH and AgNPM-AuH are shown in Figure 3D. The SERS capability of AgNPM-AuH is significantly better than that of M-AuH, which is consistent with the above analysis.

The second Ag NP deposited on M-AuH is also optimized, and Ag NPs with different morphologies are shown in Figure S2 A–C. With an increase in thickness, the size of Ag NPs becomes larger due to a decrease in gap and a metal film is formed [47, 48]. Figure 3B shows the SERS signals of R6G collected from several substrates. The Raman signal collected from the 5 nm AgNPM-AuH is much stronger than that collected from other substrates.

### 3.3 SERS performance of AgNPM-AuH with space curvatures

The AgNPM-AuH substrates possess a large space curvature with an annular groove area around the hat tip. These substrates were obtained from the initial template PS spheres with different diameters. Generally, the diameter of AgNPM-AuH is inversely proportional to the space curvature. The AgNPM-AuH arrays with sizes of 300, 700, and 900 nm were successfully constructed (Figure S3 A–C). It is worth emphasizing that the morphology of Ag NPs formed via thermal evaporation could be controlled in several substrates. Thus, the influence of the space curvature on the SERS performance of AgNPM-AuH can be investigated.

**Figure 4:**
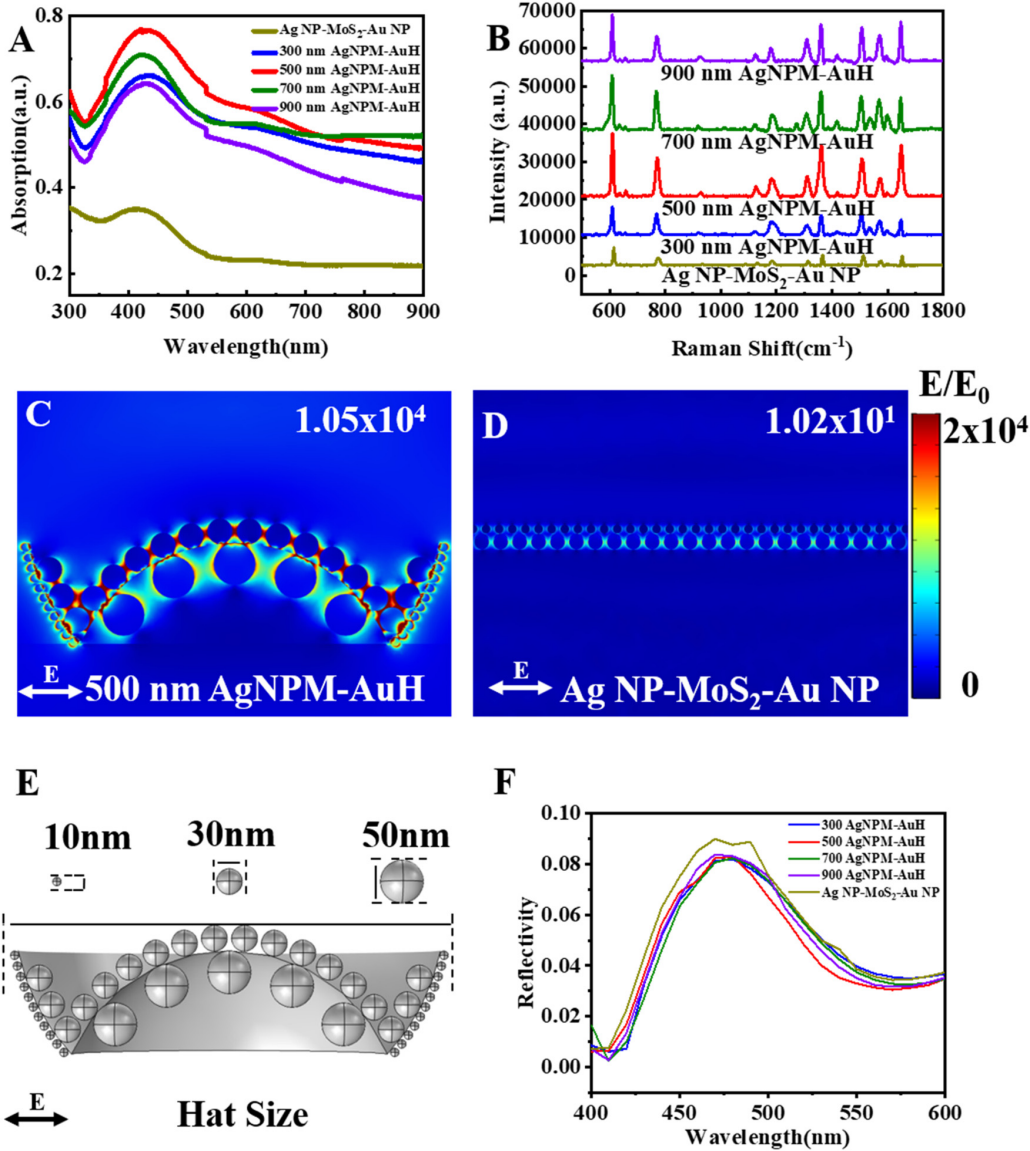
(A) Corresponding UV–vis spectroscopy of AgNPM-AuH and Ag NP/MoS_2_/Au NP substrates with various diameters. (B) Raman spectra of AgNPM-AuH and Ag NP/MoS_2_/Au NP substrates with R6G (different diameters). (C) and (D) *x* – *z* views of the electric field distribution associated with the 500 nm AgNPM-AuH substrates and Ag NP-MoS_2_ -Au NP substrates under the stimulation of 532 nm laser. The average electric field enhancement ( *E* / *E*_0_ ) is exhibited in the upper right corner. (E) Parameter setting for simulation of the AgNPM-AuH substrate. (F) The calculated reflectance spectra of the several substrates.

Figure 4A shows the UV–vis spectra of AgNPM-AuH arrays with different diameters by using the Ag NP/MoS_2_/Au NP nanostructure on a flat substrate as the reference. The light absorption of AgNPM-AuH is significantly greater than that of the Ag NP/MoS_2_/Au NP substrate. Furthermore, the hat-shaped substrate with space curvature could further localize the light energy, thereby enhancing the LSPR and obtaining a larger EM. We also found that 500-AgNPM-AuH exhibits the highest absorption intensity, thereby proving its strong light focusing ability. We further implemented a SERS experiment using above samples for a comprehensive study. Figure 4B shows that the characteristic Raman peaks of R6G can be easily distinguished; the Raman signals of R6G of AgNPM-AuH are much higher than those detected on the flat Ag NP/MoS_2_/Au NP substrate, which is in accordance with the above analysis. As expected, the 500-AgNPM-AuH still shows the best SERS performance.

To further elucidate the enhancement mechanism and the electric field distribution, the AgNPM-AuH nanostructures were theoretically simulated using COMSOL Multi-physics. In order to fabricate the simulation set-up as accurate as possible, statistics on the size of Au NP and Ag percolated film were made, respectively. The Au NP in the center is much different from that on the marginal area. Thus, the size statistics on Au NP in the two areas was carried out, respectively. We note that the average size of upper percolated Ag film was ∼30 nm with the gap ∼5 nm as shown in Figure S3 D. For the Au NP, it was found that the size of NP in the center area was ∼50 nm with ∼30 nm gap, and the smaller Au NP at the edge were ∼10 nm with ∼3 nm gap as shown in Figure S3 E–F. Based on this, the simplified unit model was fabricated and the construction parameters are shown in Figure 4E . The calculation model was extended to 1.5 um to match the actual size of the laser spot. To make the distributions of electric field be observed more clearly, the electric field enhancement of a single unit cell was taken out as shown in Figure 4C–D and Figure S4 A–C. The strongest hot spots generated on the MoS_2_ spacer area between the uplayered Ag NP and the underlying Au NP. Note that the electromagnetic field EF in AgNPM-AuH is much larger than that in the Ag NP/MoS_2_/Au NP nanostructure on a flat substrate. Previous work has proven that warped spaces can induce an effective refractive index gradient [25], which mitigates the abrupt change in the refractive index from air to the AgNPM-AuH surface. Thus, the hat-shaped structure effectively increases the optical path of the incident light, which is a critical factor to localize the exciting light.

To better elucidate the EF of the several AgNPM-AuH substrates, the reflectance spectra of the several substrates were calculated as shown in Figure 4F . It shows that the reflectance spectra of the AgNPM-AuH substrate were separated by obvious reflectivity dips around 410 and 560 nm, which should be attributed to the excitation of the LSPR of these substrates. Besides, the resonance depth is closely related to the intensity of LSPR [49]. The reflectance spectra of 500-AgNPM-AuH possess the largest resonance depth, which demonstrates the 500-AgNPM-AuH should have the strongest near-field effect. The electric field around the nanoparticles got a maximal enhancement to form largest and brightest hot spots in the 500-AgNPM-AuH as shown in Figure 4. The electric field enhancement of the warped substrates is ranked as: 300-AgNPM-AuH < 500-AgNPM-AuH > 700-AgNPM-AuH > 900-AgNPM-AuH > Ag NP/MoS_2_/Au NP, which is highly consistent with the Raman test results. The AgNPM-AuH with different diameters represents different space curvatures. Compared with other warped substrates, the space curvature in 500-AgNPM-AuH could make the greatest contribution to the improvement of the exciting light location.

### 3.4 Plasmon-induced reaction efficiency of the AgNPM-AuH

The above-mentioned experimental results and theoretical simulations confirm that the AgNPM-AuH structure possesses hot spots with high intensity and density. In general, stronger hot spots tend to generate more hot electrons via plasmonic decay, which benefits the surface catalytic reaction [50, 51]. Taking advantage of the coupling between excitons and plasmons, many studies have introduced MoS_2_ into plasmonic nanostructures to extend the presence time of hot electrons [52], [53], [54], [55]. To investigate the surface catalytic ability of AgNPM-AuH with different curvatures, *in-situ* monitoring of the dimerization reduction reaction of PNTP to DMAB was conducted. To ensure that the PNTP molecules are uniformly distributed throughout the structure, the AgNPM-AuH substrate is immersed in a 10^−3^ M PNTP ethanol solution for 90 min and dried in N_2_ atmosphere after several alcohol washes.

**Figure 5:**
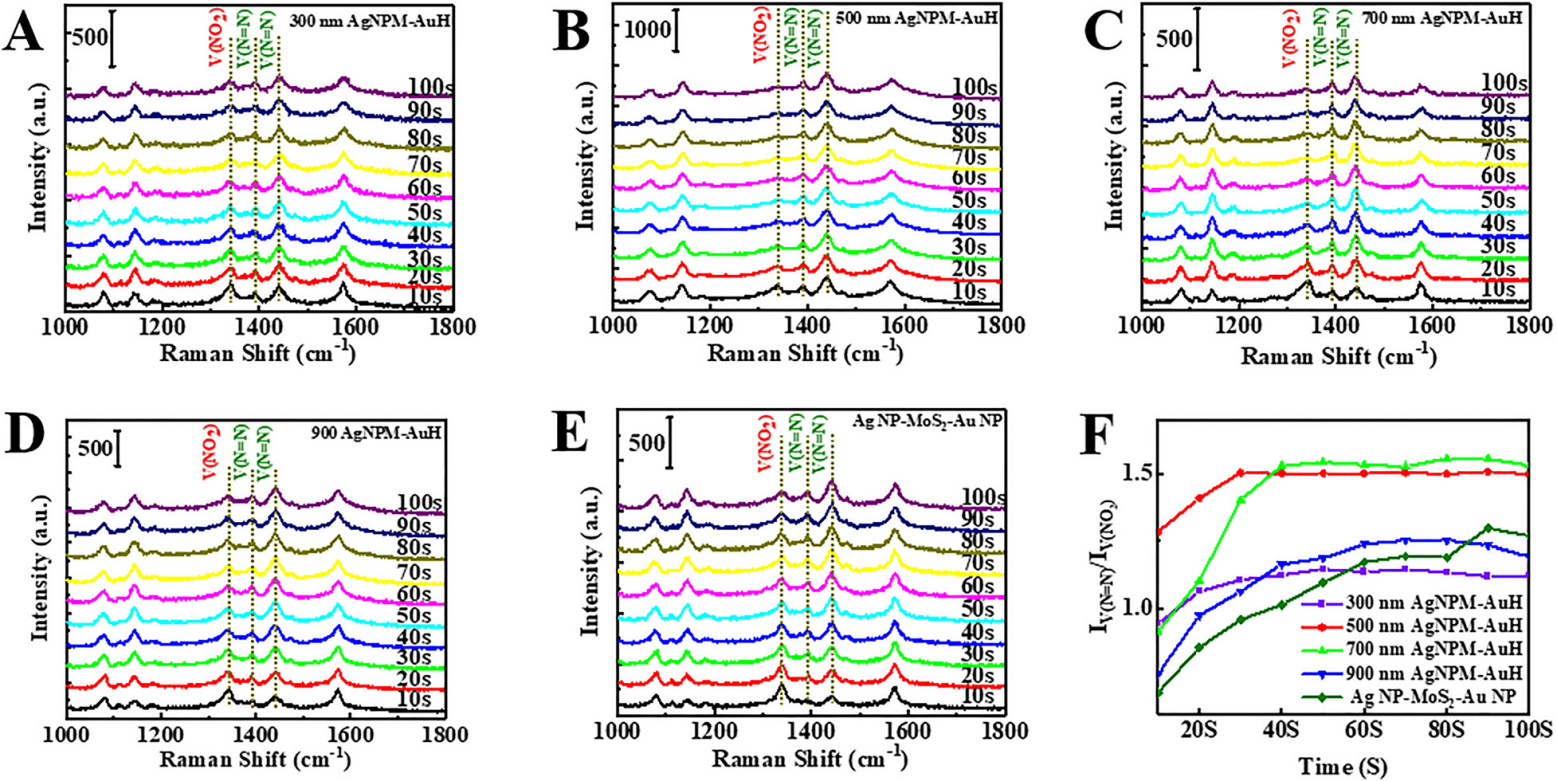
(A)–(E) Bifunctional catalytic activity and *in situ* SERS monitoring of the dimerization of PNTP to DMAB for AgNPM-AuH and Ag NP-M-Au NP substrates with various diameters. (F) Variation in Raman intensity ratio with time for *v*(N=N) and *v*(NO_2_) bands for various diameters of AgNPM-AuH.

As demonstrated in Figure 5A–E, the decreasing *ν*(NO_2_) peak of PNTP at 1340 cm^−1^ and the newly formed *ν*(N=N) peaks of DMAB at 1397 and 1445 cm^−1^ represent the conversion of PNTP to DMAB [56, 57]. The *ν*(N=N) peak becomes stronger and the *ν*(NO_2_) peak gradually decreases after the initiation of the reaction, indicating the constant production of DMAB. The time-dependent Raman intensity ratio $I_{\nu \left(\text{N}=\text{N}\right)}/I_{\nu \left({\text{NO}}_{2}\right)}$ can be used to estimate reaction rate in Figure 5F [58, 59], where *I*_*v*(N=N)_ represents the 1445 cm^−1^ DMAB band and $I_{\nu \left({\text{NO}}_{2}\right)}$ denotes the 1340 cm^−1^ PNTP band. Generally, a shorter time period required to reach reaction equilibrium indicates a stronger catalytic ability.

We found that the 500-AgNPM-AuH substrate exhibits the best catalytic ability by reaching an equilibrium state within 30 s of the reaction, with the PNTP peak completely disappearing. Meanwhile, the final *ν*(NO_2_) peak is still higher than the *ν*(N=N) peak after nearly 100 s of reaction on the Ag NP/MoS_2_/Au NP nanostructure placed on a flat substrate. The catalytic capacity of the obtained substrates is ranked as: 500-AgNPM-AuH > 700-AgNPM-AuH > 300-AgNPM-AuH > 900-AgNPM-AuH > Ag NP/MoS_2_/Au NP; this is consistent with the electromagnetic field intensity observed via simulation. Considering the fact that the hot electrons induce the reduction process, the substrates with stronger plasmon coupling ought to generate more hot electrons to accelerate the reaction process [60], [61], [62]. Furthermore, the Raman signal intensity of DMAB derived from 500-AgNPM-AuH is stronger than that sourced from other substrates, which is in accordance with the above-mentioned experimental results. Furthermore, the AgNPM-AuH structure could significantly increase the SERS effect and facilitate the surface catalytic reaction due to the nanoparticle-gap-nanoparticle model and the space curvature effect.

### 3.5 SERS performance of the AgNPM-AuH SERS substrates

High sensitivity, quantitative determination, reproducibility, and stability are crucial for an excellent SERS performance; accordingly, we verify the applicability of the AgNPM-AuH substrate for SERS.

**Figure 6:**
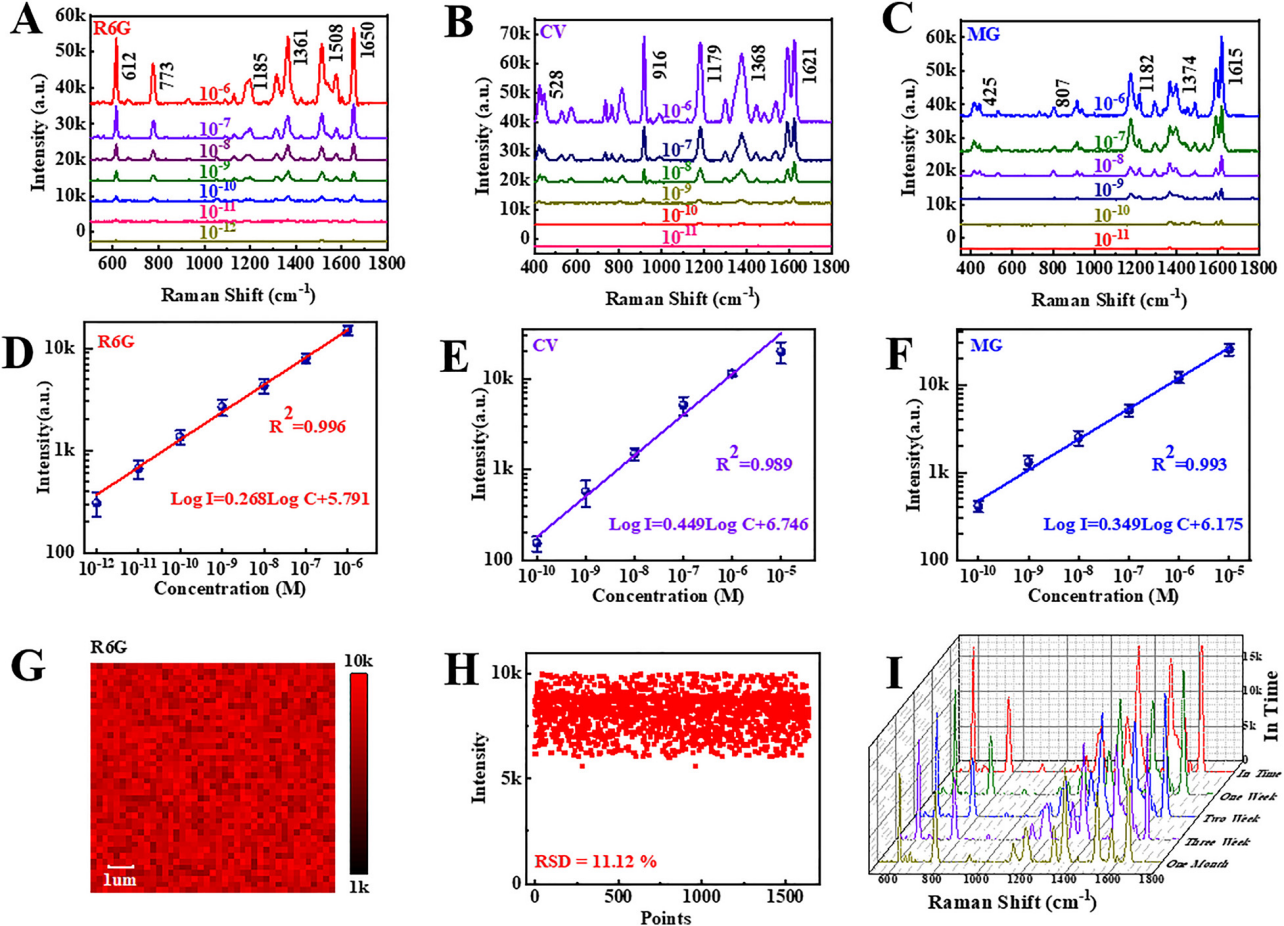
(A)–(C) SERS spectra of R6G, CV, and MG at varying concentrations. (D)–(F) Linear fitting lines of R6G, CV, and MG. (G) Raman mapping of R6G (10 ^−6^ M) collected from 900 nm AgNPM-AuH. (H) The RSD values of 612 cm ^−1^ bands collected from 900 nm AgNPM-AuH. (I) Raman spectra of R6G derived from the AgNPM-AuH substrate for one month.

(1) High sensitivity: CV, MG, and R6G are the three common contaminants. Figure 6A shows the various R6G concentrations (10^−12^–10^−6^ M) and Raman signals derived from the substrates; the lowest detectable concentration reaches 10^−12^ M at a single molecule detection level. AgNPM-AuH substrates also exhibit an excellent trace detection capability for other molecules shown in Figure 6B and C. The detection limits for CV and MG reach 10^−11^ M, indicating the general applicability of the SERS substrates and demonstrating that the AgNPM-AuH substrate possesses high Raman sensitivity.
(2) Quantitative determination: We selected the Raman intensity at the 612 cm^−1^ peak to elucidate the relationship between intensity (log *I*) and concentration of R6G (log *C*) (Figure 6D). The linear relationship expresses the variation in intensity within the concentration as log *I* = 0.268Log *C* + 5.791, and the determination coefficient (*R* ^2^ ≈ 0.996) shows an excellent linear relation. Subsequently, we selected the 916 cm^−1^ peak of CV to obtain a linear fitting line shown in Figure 6E (expressed as log *I* = 0.449Log *C* + 6.746; *R* ^2^ is 0.989). Similarly, the 1621 cm^−1^ peak represents the MG molecules detected within the same concentration range (Log *I* = 0.349Log *C* + 6.175; *R* ^2^ is 0.993. This indicates that the AgNPM-AuH substrate could achieve a good quantitative analysis for probe molecules.
(3) Reproducibility: To verify the repeatability of AgNPM-AuH, the signals of 10 ^−6^ M R6G are measured randomly and the mapping spectra are obtained (Figure S4 D–E). The peak intensities of all the spectra are almost identical, which exhibit the excellent reproducibility of AgNPM-AuH. In addition to high sensitivity, high hot spot density has been proven to be closely related to signal reproducibility [46, 63]. In addition, the mapping spectra of R6G were also collected from the 900 nm AgNPM-AuH structure (Figure 6G), and the relative standard deviation (RSD%) is calculated to be 11.12% (Figure 6H) demonstrating the high homogeneity of the larger periodic structure.
(4) Stability: Stability is extremely important in practical application scenarios. However, the upper Ag NPs of the substrate are prone to oxidation under normal atmospheric conditions, which can decrease the SERS signals. The formation of Ag_2_O under laser irradiation in the SERS test should contribute to the stability of Ag NPs [64, 65]. Furthermore, the strongest local electric field is generated on the MoS_2_ spacer area between the upper Ag NPs and the underlying Au NPs, which provides the greatest contribution to the total SERS signals. The Ag NPs are in close contact with MoS_2_, which protects the bottom surface of the Ag NPs from oxidation to some extent. Thus, the attenuation of measured Raman signals is relatively small (Figure 6I). The 612 cm^−1^ peak decreased by 28.7% during one month, which demonstrates the good stability of AgNPM-AuH.

To intuitively evaluate the SERS effect of AgNPM-AuH, the enhancement factor can be expressed by calculating the following equation [20, 28, 66]:

$$\text{EF}=\frac{I_{SERS}\times N_{RS}}{I_{RS}\times N_{SERS}}$$

*I*_SERS_ and *I*_RS_ represent the peak intensities of the SERS substrate and Raman signals on SiO_2_. *N*_SERS_ and *N*_RS_ represent the number of probe molecules on SERS substrate and SiO_2_. The 10^−12^ M R6G cannot be detected anywhere in the substrates for extremely sparse molecules, and the SERS signal of the 10^−10^ M R6G was considered for EF. By using the 10^−2^ M R6G solution dropped on SiO_2_ for comparison, the best substrate achieved an EF of 1.09 × 10^9^ for R6G of 500-AgNPM-AuH, which was much higher than that reported for other SERS substrates (Table 1).

**Table 1:** Comparison between the reported MoS_2_ hybrid nanostructures and the AgNPM-AuH substrate.

Structure	The material composition	EF	Reference
Few-layer MoS_2_	MoS_2_ film	7.68 × 10^2^	[67]
1T-MoS_2_/AgNCs	1T-MoS_2_ nanosheets decorated with silver nanocubes	1.78 × 10^7^	[68]
PSi/MoS_2_/Au MSC	Pyramid Si,MoS_2_, and Au NPs	6.5 × 10^7^	[69]
MoS_2_ nanoflowers/AuNPs	MoS_2_ nanoflowers, AuNPs	1.69 × 10^7^	[70]
ID-MoS_2_ nanosheets	ID-MoS_2_ nanosheets	1.24 × 10^7^	[71]
1T-2H MoS_2_/Au heterostructure	1T-2H MoS_2_, Au NPs	8.1 × 10^6^	[72]
AuNPs/GO@MoS_2_/AuNPs	AuNPs, GO, MoS_2_, and Ag NPs	3.64 × 10^8^	[73]
AgNPM-AuH	Ag NPs, MoS_2_ hat, and Au NPs	1.09 × 10^9^	This work

## 4 Conclusions

In summary, a highly ordered nanostructure with two-layered metal nanoparticles separated by hat-shaped MoS_2_ films was proposed. Combined with theoretical simulations, it was proven that apart from the high-intensity hot spots generated by the sandwich structures, the space curvature could further enhance the light absorption and enhance the local electromagnetic field. The SERS performance of the proposed AgNPM-AuH substrates with different curvatures were also investigated. The substrates achieved low detection limits for common contaminants like R6G, CV, and MG. Furthermore, the AgNPM-AuH substrate also demonstrated excellent catalytic performance and its catalytic ability has been closely related to the space curvature. We anticipate that the bifunctional AgNPM-AuH substrate will be widely used for SERS detection and proton-driven photocatalytic applications.

## Supplementary Material

Supplementary Material

## References

[1] Lyon L. A., Keating C. D., Fox A. P. (1998). Raman spectroscopy. *Anal. Chem.*.

[2] Nie S., Emory S. R. (1997). Probing single molecules and single nanoparticles by surface-enhanced Raman scattering. *Science*.

[3] Chen R. P., Du X., Cui Y. J. (2020). Vertical flow assay for inflammatory biomarkers based on nanofluidic channel array and SERS nanotags. *Small*.

[4] Hassan M. M., Zareef M., Xu Y. (2021). SERS based sensor for mycotoxins detection: challenges and improvements. *Food Chem.*.

[5] Xu K., Zhou R., Takei K. (2019). Toward flexible surface‐enhanced Raman scattering (SERS) sensors for point‐of‐care diagnostics. *Adv. Sci.*.

[6] Le Ru E. C., Blackie E., Meyer M. (2007). Surface enhanced Raman scattering enhancement factors: a comprehensive study. *J. Phys. Chem. C*.

[7] Fang Y., Seong N. H., Dlott D. D. (2008). Measurement of the distribution of site enhancements in surface-enhanced Raman scattering. *Science*.

[8] Schlücker S. (2014). Surface‐enhanced Raman spectroscopy: concepts and chemical applications. *Angew. Chem. Int. Ed.*.

[9] Li C. H., Xu S. C., Yu J. (2021). Local hot charge density regulation: vibration-free pyroelectric nanogenerator for effectively enhancing catalysis and *in-situ* surface enhanced Raman scattering monitoring. *Nano Energy*.

[10] Li M., Wei Y., Fan X. (2021). Mixed-dimensional van der Waals heterojunction-enhanced Raman scattering. *Nano Res.*.

[11] Talley C. E., Jackson J. B., Oubre C. (2005). Surface-enhanced Raman scattering from individual Au nanoparticles and nanoparticle dimer substrates. *Nano Lett.*.

[12] Yoon I., Kang T., Choi W. (2009). Single nanowire on a film as an efficient SERS-active platform. *J. Am. Chem. Soc.*.

[13] Lee A., Andrade G. F. S., Ahmed A. (2011). Probing dynamic generation of hot-spots in self-assembled chains of gold nanorods by surface-enhanced Raman scattering. *J. Am. Chem. Soc.*.

[14] Chen H. Y., Lin M. H., Wang C. Y. (2015). Large-scale hot spot engineering for quantitative SERS at the single-molecule scale. *J. Am. Chem. Soc.*.

[15] Wang P., Liang O., Zhang W. (2013). Ultra‐sensitive graphene‐plasmonic hybrid platform for label‐free detection. *Adv. Mater.*.

[16] Li J. F., Huang Y. F., Ding Y. (2010). Shell-isolated nanoparticle-enhanced Raman spectroscopy. *Nature*.

[17] Fateixa S., Nogueira H. I. S., Trindade T. (2015). Hybrid nanostructures for SERS: materials development and chemical detection. *Phys. Chem. Chem. Phys.*.

[18] Zhan Z. Y., Liu L. H., Wang W. (2016). Ultrahigh surface-enhanced Raman scattering of graphene from Au/graphene/Au sandwiched structures with subnanometer gap. *Adv. Opt. Mater.*.

[19] Kim H., Seol M. L., Lee D. I. (2016). Single nanowire on graphene (SNOG) as an efficient, reproducible, and stable SERS-active platform. *Nanoscale*.

[20] Park S. G., Mun C., Lee M. (2015). 3D hybrid plasmonic nanomaterials for highly efficient optical absorbers and sensors. *Adv. Mater.*.

[21] Novikov S. M., Boroviks S., Evlyukhin A. B. (2020). Fractal shaped periodic metal nanostructures atop dielectric-metal substrates for SERS applications. *ACS Photonics*.

[22] Tian Y., Wang H. F., Yan L. Q. (2019). A generalized methodology of designing 3D SERS probes with superior detection limit and uniformity by maximizing multiple coupling effects. *Adv. Sci.*.

[23] Yu J., Guo Y., Wang H. J. (2019). Quasi optical cavity of hierarchical ZnO nanosheets@Ag nanoravines with synergy of near- and far-field effects for *in situ* Raman detection. *J. Phys. Chem. Lett.*.

[24] Li X. L., Zhang Y. Z., Shen Z. X. (2012). Highly ordered arrays of particle-in-bowl plasmonic nanostructures for surface-enhanced Raman scattering. *Small*.

[25] Mao P., Liu C. X., Favraud G. (2018). Broadband single molecule SERS detection designed by warped optical spaces. *Nat. Commun.*.

[26] Li Z., Li C. H., Yu J. (2020). Aluminum nanoparticle films with an enhanced hot-spot intensity for high-efficiency SERS. *Opt. Express*.

[27] Ling X., Fang W. J., Lee Y. H. (2014). Raman enhancement effect on two-dimensional layered materials: graphene, h-BN and MoS_2_. *Nano Lett.*.

[28] Ye J., Bonroy K., Nelis D. (2008). Enhanced localized surface plasmon resonance sensing on three-dimensional gold nanoparticles assemblies. *Colloid. Surface. Physicochem. Eng. Aspect.*.

[29] Lin Y., Zhang Y. J., Yang W. M. (2019). Size and dimension dependent surface-enhanced Raman scattering properties of well-defined Ag nanocubes. *Appl. Mater. Today*.

[30] Hamon C., Novikov S. M., Scarabelli L. (2015). Collective plasmonic properties in few-layer gold nanorod supercrystals. *ACS Photonics*.

[31] Anderson W. J., Nowinska K., Hutter T. (2018). Tuning plasmons layer-by-layer for quantitative colloidal sensing with surface-enhanced Raman spectroscopy. *Nanoscale*.

[32] Pendry J. B., Fernandez-Dominguez A. I., Luo Y. (2013). Capturing photons with transformation optics. *Nat. Phys.*.

[33] Luo Y., Pendry J. B., Aubry A. (2010). Surface plasmons and singularities. *Nano Lett.*.

[34] Galinski H., Favraud G., Dong H. (2017). Scalable, ultra-resistant structural colors based on network metamaterials. *Light Sci. Appl.*.

[35] Li L., Liu C., Cao X. W. (2017). Determination of carcinoembryonic antigen by surface-enhanced Raman spectroscopy using gold nanobowl arrays. *Anal. Lett.*.

[36] Hovel M., Gompf B., Dressel M. (2010). Dielectric properties of ultrathin metal films around the percolation threshold. *Phys. Rev. B*.

[37] Li Z., Jiang S. Z., Huo Y. Y. (2018). 3D hybrid plasmonic nanostructures with dense hot spots using monolayer MoS_2_ as sub-nanometer spacer. *Adv. Mater. Interfaces*.

[38] Chen B. S., Meng G. W., Zhou F. (2014). Ordered arrays of Au-nanobowls loaded with Ag-nanoparticles as effective SERS substrates for rapid detection of PCBs. *Nanotechnology*.

[39] Yang X. Z., Yu H., Guo X. (2017). Plasmon-exciton coupling of monolayer MoS_2_-Ag nanoparticles hybrids for surface catalytic reaction. *Mater. Today Energy*.

[40] Shen Y. F., Miao P., Hu C. (2018). SERS-based plasmon-driven reaction and molecule detection on a single Ag@MoS_2_ microsphere: effect of thickness and crystallinity of MoS_2_. *ChemCatChem*.

[41] Lee C., Yan H., Brus L. E. (2010). Anomalous lattice vibrations of single- and few-layer MoS_2_. *ACS Nano*.

[42] Zhou K. G., Withers F., Cao Y. (2014). Raman modes of MoS_2_ used as fingerprint of van der Waals interactions in 2-D crystal-based heterostructures. *ACS Nano*.

[43] Plechinger G., Heydrich S., Eroms J. (2012). Raman spectroscopy of the interlayer shear mode in few-layer MoS_2_ flakes. *Appl. Phys. Lett.*.

[44] Mao N. N., Chen Y. F., Liu D. M. (2013). Solvatochromic effect on the photoluminescence of MoS_2_ monolayers. *Small*.

[45] Conley H. J., Wang B., Ziegler J. I. (2013). Bandgap engineering of strained monolayer and bilayer MoS_2_. *Nano Lett.*.

[46] Gong C., Huang C. M., Miller J. (2013). Metal contacts on physical vapor deposited monolayer MoS. *ACS Nano*.

[47] Yakubovsky D. I., Stebunov Y. V., Kirtaev R. V. (2019). Ultrathin and ultrasmooth gold films on monolayer MoS_2_. *Adv. Mater. Interfaces*.

[48] Tatarkin D. E., Yakubovsky D. I., Ermolaev G. A. (2020). Surface-enhanced Raman spectroscopy on hybrid graphene/gold substrates near the percolation threshold. *Nanomaterials*.

[49] Wu H. Y., Choi C. J., Cunningham B. T. (2012). Plasmonic nanogap-enhanced Raman scattering using a resonant nanodome array. *Small*.

[50] Cui L., Li R., Mu T. (2022). *In situ* plasmon-enhanced CARS and TPEF for gram staining identification of non-fluorescent bacteria. *Spectrochim. Acta Mol. Biomol. Spectrosc.*.

[51] Zhang C. P., Chen S., Jiang Z. L. (2021). Highly sensitive and reproducible SERS substrates based on ordered micropyramid array and silver nanoparticles. *ACS Appl. Mater. Interfaces*.

[52] Xu P., Kang L. L., Mack N. H. (2013). Mechanistic understanding of surface plasmon assisted catalysis on a single particle: cyclic redox of 4-aminothiophenol. *Sci. Rep.*.

[53] Lantman E. M. V., Gijzeman O. L. J., Mank A. J. G. (2014). Investigation of the kinetics of a surface photocatalytic reaction in two dimensions with surface-enhanced Raman scattering. *ChemCatChem*.

[54] Lal S., Grady N. K., Kundu J. (2008). Tailoring plasmonic substrates for surface enhanced spectroscopies. *Chem. Soc. Rev.*.

[55] Kang Y. M., Najmaei S., Liu Z. (2014). Plasmonic hot electron induced structural phase transition in a MoS_2_ monolayer. *Adv. Mater.*.

[56] Zhao W. J., Wang S. F., Liu B. (2016). Exciton-plasmon coupling and electromagnetically induced transparency in monolayer semiconductors hybridized with Ag nanoparticles. *Adv. Mater.*.

[57] Zhao L. B., Zhang M., Huang Y. F. (2014). Theoretical study of plasmon-enhanced surface catalytic coupling reactions of aromatic amines and nitro compounds. *J. Phys. Chem. Lett.*.

[58] Wang Y., Chen L. Y., Xu B. (1998). The medium-related optical constants of noble metals observed by ellipsometric study. *Thin Solid Films*.

[59] Fang Y. R., Li Y. Z., Xu H. X. (2010). Ascertaining p,p’-dimercaptoazobenzene produced from p-aminothiophenol by selective catalytic coupling reaction on silver nanoparticles. *Langmuir*.

[60] Zu S., Li B. W., Gong Y. J. (2016). Active control of plasmon-exciton coupling in MoS_2_-Ag hybrid nanostructures. *Adv. Opt. Mater.*.

[61] Zheng Z. K., Tachikawa T., Majima T. (2015). Plasmon-enhanced formic acid dehydrogenation using anisotropic Pd–Au nanorods studied at the single-particle level. *J. Am. Chem. Soc.*.

[62] Xie W., Schlucker S. (2015). Hot electron-induced reduction of small molecules on photorecycling metal surfaces. *Nat. Commun.*.

[63] Zhai W. L., Li D. W., Qu L. L. (2012). Multiple depositions of Ag nanoparticles on chemically modified agarose films for surface-enhanced Raman spectroscopy. *Nanoscale*.

[64] Chen H. L., Yang Z. H., Lee S. (2016). Observation of surface coverage-dependent surface-enhanced Raman scattering and the kinetic behavior of methylene blue adsorbed on silver oxide nanocrystals. *Langmuir*.

[65] Li C. H., Yang C., Xu S. C. (2017). Ag_2_O@Ag core-shell structure on PMMA as low-cost and ultra-sensitive flexible surface-enhanced Raman scattering substrate. *J. Alloys Compd.*.

[66] Kneipp K., Kneipp H., Itzkan I. (1999). Ultrasensitive chemical analysis by Raman spectroscopy. *Chem. Rev.*.

[67] Majee B. P., Mishra S., Pandey R. K. (2019). Multifunctional few-layer MoS_2_ for photodetection and surface-enhanced Raman spectroscopy application with ultrasensitive and repeatable detectability. *J. Phys. Chem. C*.

[68] Tegegne W. A., Su W. N., Tsai M. C. (2020). Ag nanocubes decorated 1T-MoS_2_ nanosheets SERS substrate for reliable and ultrasensitive detection of pesticides. *Appl. Mater. Today*.

[69] Zhao X. F., Liu C. D., Yu J. (2020). Hydrophobic multiscale cavities for high-performance and self-cleaning surface-enhanced Raman spectroscopy (SERS) sensing. *Nanophotonics*.

[70] Sun H. H., Yao M. G., Song Y. P. (2019). Pressure-induced SERS enhancement in a MoS_2_/Au/R6G system by a two-step charge transfer process. *Nanoscale*.

[71] Zhou X. Y., Wu D., Jin Z. (2020). Significantly increased Raman enhancement on defect-rich O-incorporated 1T-MoS(2) nanosheets. *J. Mater. Sci.*.

[72] Zheng X. L., Guo Z. H., Zhang G. Y. (2019). Building a lateral/vertical 1T-2H MoS_2_/Au heterostructure for enhanced photoelectrocatalysis and surface enhanced Raman scattering. *J. Mater. Chem. A*.

[73] Zhang H., Zhang W. Y., Gao X. (2019). Formation of the AuNPs/GO@MoS_2_/AuNPs nanostructures for the SERS application. *Sensor. Actuator. B Chem.*.

