# Supplementary material for: Highly ordered arrays of hat-shaped hierarchical nanostructures with different curvatures for sensitive SERS and plasmon-driven catalysis
Source: Nanophotonics. 2021 Nov 15;11(1):33–44. doi: 10.1515/nanoph-2021-0476 (PMC11501450; doi:10.1515/nanoph-2021-0476)
Supplement: Supplementary file 1 — Supplementary Material [file j_nanoph-2021-0476_suppl_001.docx]

# Highly Ordered Arrays of hat-shaped hierarchical nanostructures with different curvature for sensitive SERS and plasmon-driven catalysis

Chao Zhang^a^, Zhaoxiang Li^a^, Si Qiu ^a^,Weixi Lu^a^, Mingrui Shao^a^, Chang Ji^a^, Guangcan Wang^a^, Xiaofei Zhao^a^, Jing Yu^a,^*, and Zhen Li^a,^*^[[1]](#footnote-1)^

^a^ School of Physics and Electronics, Shandong Normal University, Jinan 250014, China


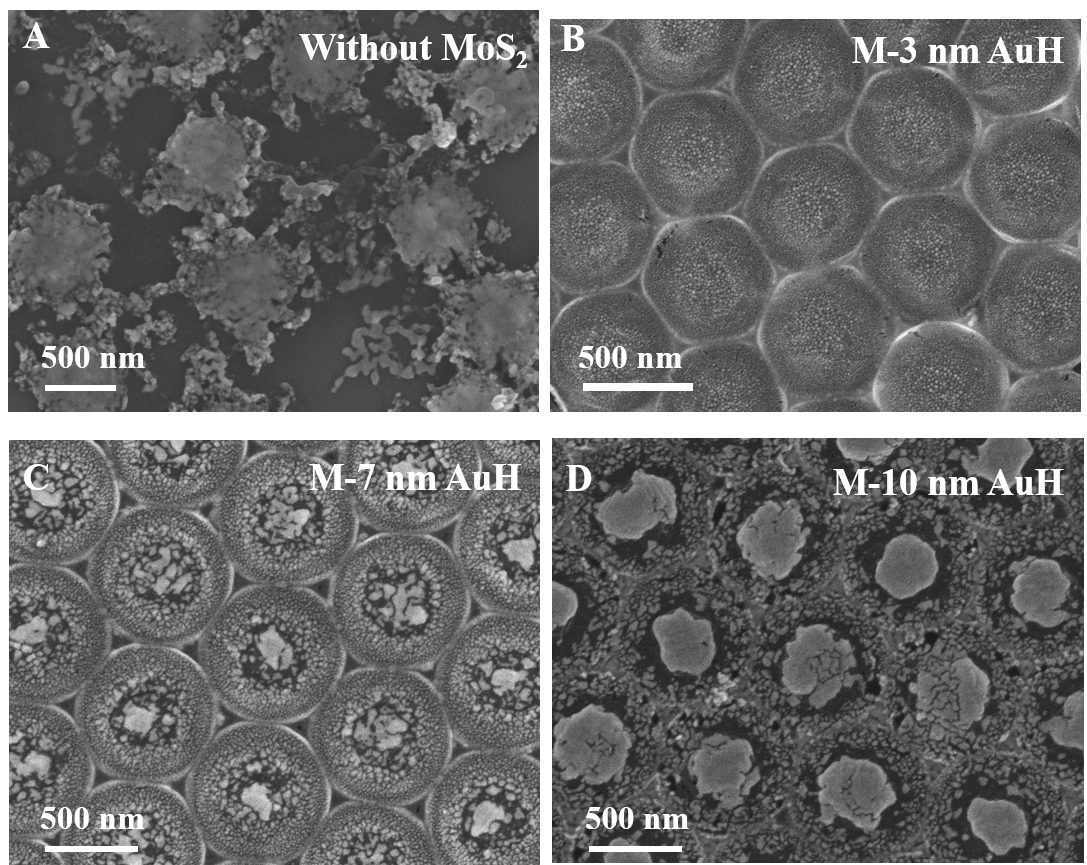


Fig S1 (A) PS sphere covered percolated Au film grow without (NH_4_)_2_MoS_4_. SEM images of the different thickness of Au film: (B)3 nm, (C)7 nm, (D)10 nm.


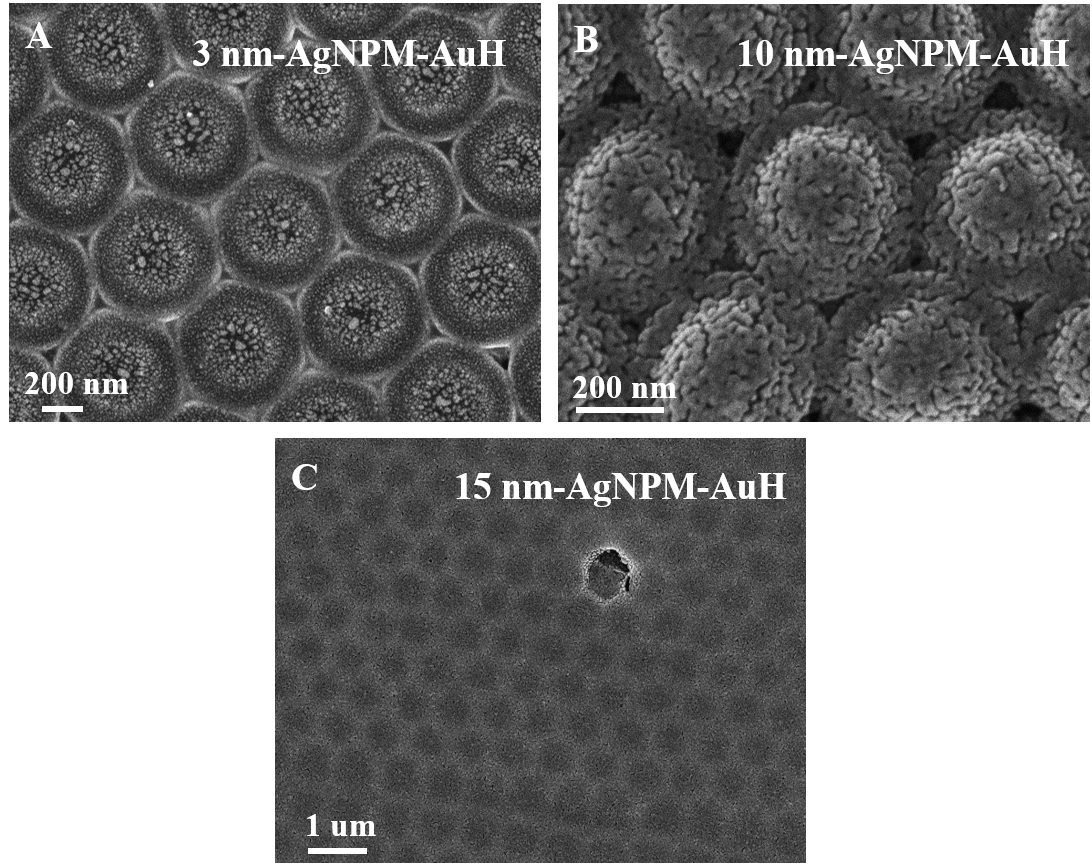


Fig. S2. (A)-(C) are respectively the SEM characterizations of the M-AuH covered with different thickness of percolated Ag film: 3 nm, 10 nm,15 nm.


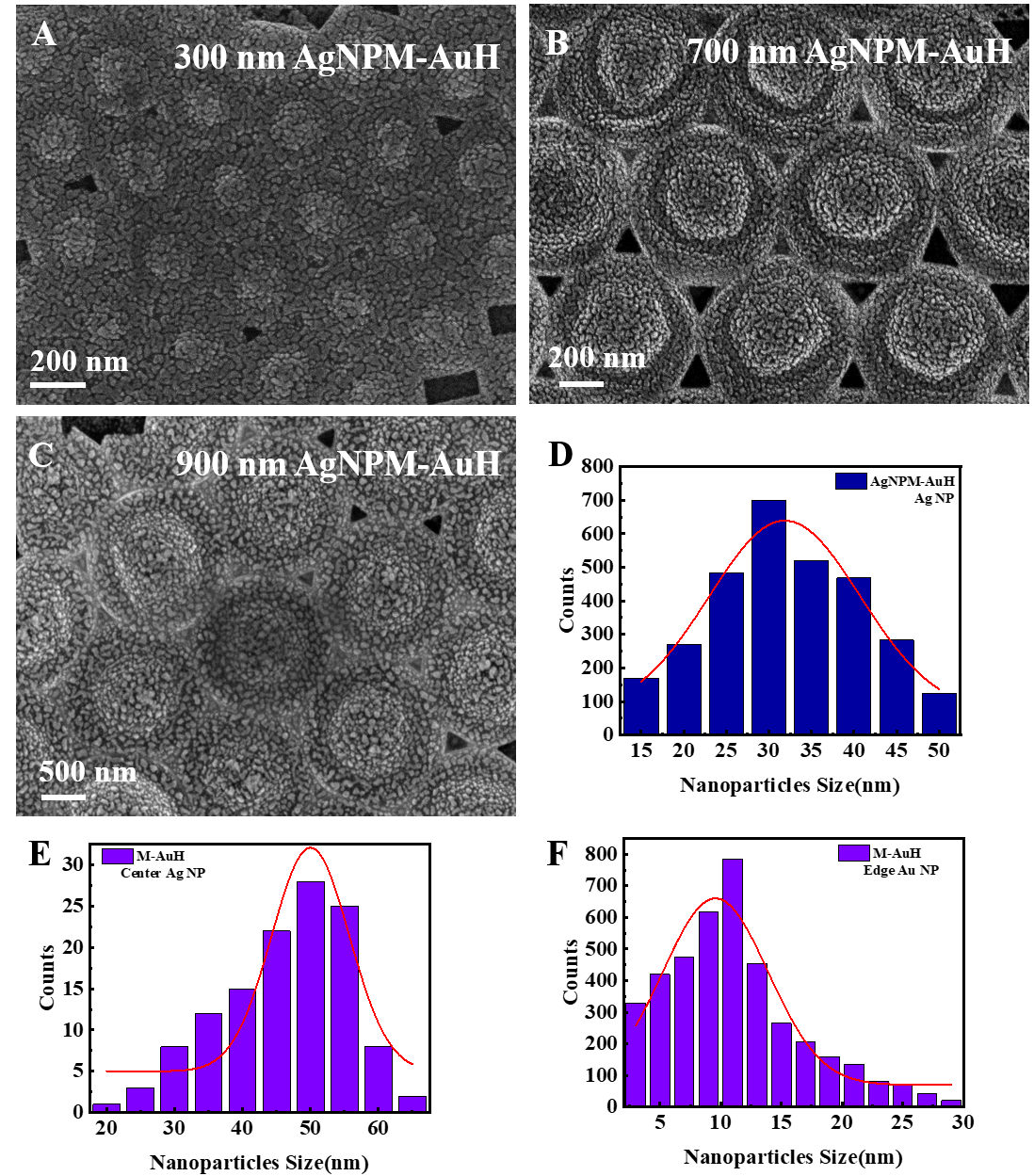


Fig. S3. (A)-(C) are respectively the SEM characterizations of the AgNPM-AuH fabricated by different diameter of PS sphere: 300 nm, 700 nm, 900 nm. (D) The size of the Ag NP on AgNPM-AuH. (E)-(F) The size of the Au NP on M-AuH center and edge,


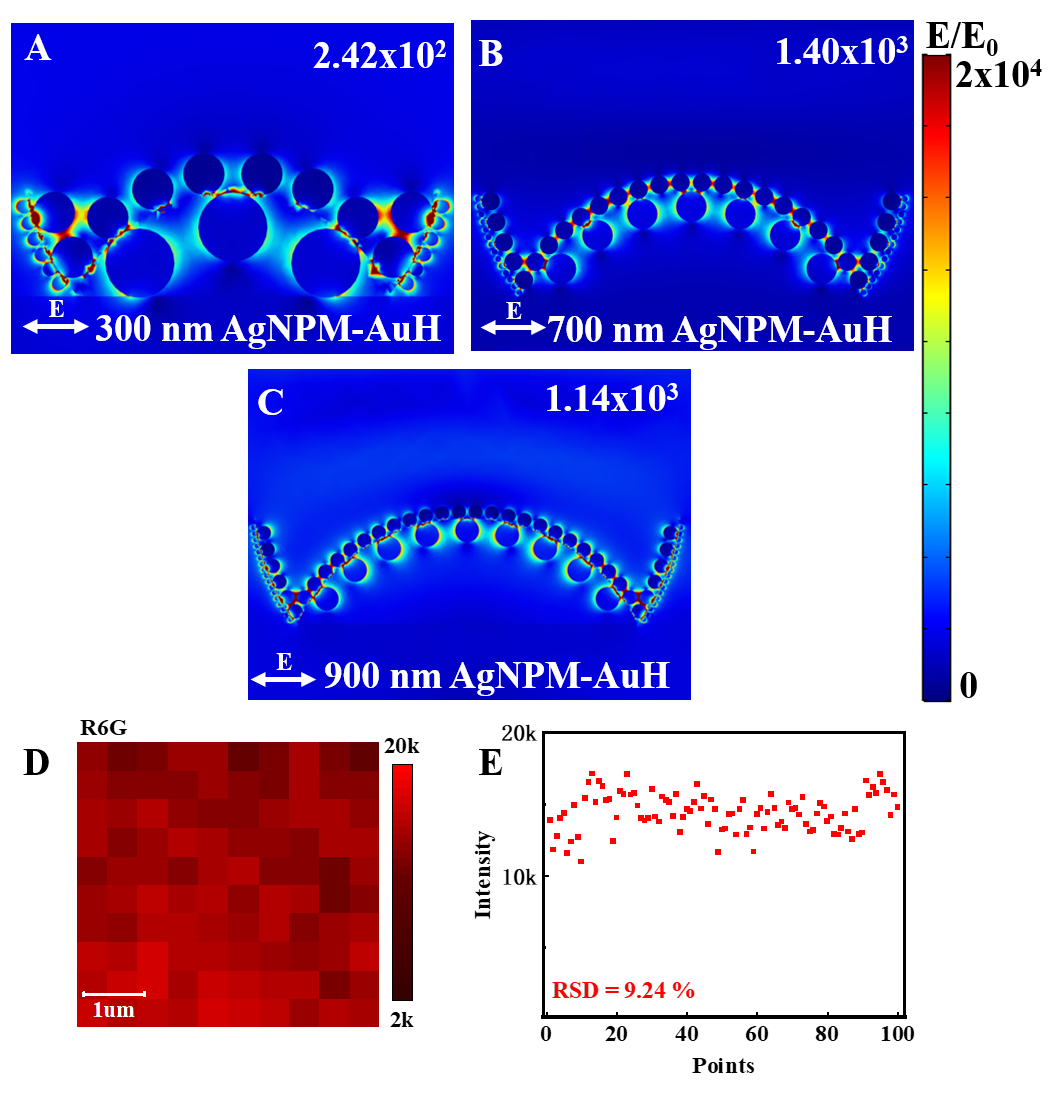


Fig. S4. (A)-(C) x–z views of the electric field distribution associated with the AgNPM-AuH and Ag NP/MoS_2_/Au NP substrates with different diameters at 532 nm. Electric field direction marked out and the average electric field strength is indicated in the upper right corner. (D)-(E) The Raman mapping of R6G (10^-6^ M) was obtained on a 500 nm AgNPM-AuH substrate with 5 um side length and 100 points scanned, and the RSD value.

1. * Corresponding author.

   E-mail: yujing1608@126.com and lizhen19910528@163.com (Zhen Li). [↑](#footnote-ref-1)
